# High–efficiency generation of induced pluripotent stem cells from human foreskin fibroblast cells using the Sagunja-tang herbal formula

**DOI:** 10.1186/s12906-017-2043-2

**Published:** 2017-12-11

**Authors:** Ki Mo Kim, Deok Rim Heo, Joo Young Lee, Chang-Seob Seo, Sun-Ku Chung

**Affiliations:** 1 KM Convergence Research Division, Daejeon, Republic of Korea; 2 K-herb Research Center, Daejeon, Republic of Korea; 3 Mibyeong Research Center, Korea Institute of Oriental Medicine, 1672 Yuseong-daero, Yuseong-gu, Daejeon, 34054 Republic of Korea; 4 Korean Life Science and Technology, University of Science and Technology, Daejeon, 34134 Republic of Korea; 5 Mibyeong Research Center, Korea Institute of Oriental Medicine, 1672 Yuseong-daero, Yuseong-gu, Daejeon, 34054 Republic of Korea

**Keywords:** Sagunja-tang, Induced pluripotent stem cells, Reactive oxygen species, Human foreskin fibroblast, Alkaline phosphatase staining

## Abstract

**Background:**

Sagunja-Tang (SGT-4) is a traditional herbal formula in Korean medicine that is used to treat anti-metabolic syndrome, and has antioxidant activity. In this study, we evaluated the effects of SGT-4 on the formation efficiency of induced pluripotent stem cells (iPSCs) from human foreskin fibroblasts (HFFs) by four reprogramming transcription factors: Oct4, Sox2, KIf4, and c-Myc (OSKM).

**Methods:**

SGT-4 contained four different herbal medicines that are composed of *Ginseng Radix*, *Glycyrrhizae Radix et Rhizoma*, *Atractylodis Rhizoma Alba*, and *Poria Sclerotium.* The composition of SGT-4 was analyzed by high-performance liquid chromatography (HPLC). HFFs were transfected with episomal vectors contained by four OSKM. Western blotting, RT-PCR, immunofluroescence, and *in vitro* differentiation were used to assess the pluripotency of the iPSC cells.

**Results:**

SGT-4 exhibited antioxidant activity against the generation of intracellular reactive oxygen species (ROS) as well as promoted the activation of superoxide dismutase 1 (SOD1), catalase, gluthathione peroxidase 1 (GPX1), and glutathione (GSH). Moreover, the ATP level was not significantly fluctuated depending on the concentration of SGT-4 in the hiPSCs.

**Conclusion:**

Our results indicate that the SGT-4, herbal formula significantly increases the efficiency of human iPSC generation via the transcription factors (Oct4, Sox2, KIf4, and c-Myc).

## Background

Induced pluripotent stem cells (iPSCs) are an attractive potential resource for autologous cell replacement therapy. Thus, it is necessary to still improve the efficiency of cellular reprogramming, which is currently one of the major issues in iPSC field. Because of the infinite proliferative abilities and self-renewal capacity of iPSCs, these cells cannot only provide limitless amounts of cellular therapeutic materials, but also potentially differentiate toward any cell types within our bodies.

According to previous studies, the p53 protein has a negatively affects reprogramming efficiency by intrinsically inducing either apoptosis or cell cycle arrest. Thus, only temporary knockdown of p53 function can effectively improve the reprogramming efficiency [1]. In addition to genetic factors, several chemicals that can influence on chromatic structure, can also affect the reprogramming process positively. Most reported chemicals often modulate the chromatin structure and gene activity in the entire genome [2, 3]. For example, recent studies have demonstrated that valproic acid (VPA), a histone deacetylase inhibitor, has been shown to improve both the kinetics and efficiency of mouse and human iPSC generation [2]. In addition, BIX-01294, an inhibitor of histone methyltransferase G9a, and RG108, a DNA methyltransferase inhibitor, have been reported to enhance the efficiency of iPSC generation [4, 5].

Recently, butyrate, a naturally occurring short chain fatty acid and histone deacetylase inhibitor, has been shown to support the self-renewal of both human and mouse embryonic stem (ES) cells within a range of low concentrations relatively [6], whereas higher concentration of butyrate have also been reported to induce differentiation [7]. In addition, efforts to improve iPSCs safety have focused on using fewer factors and non-vital methods. For example, it was reported that Oct4 alone could reprogram human epidermal keratinocytes into human iPSCs (hiPSCs) [8].

Sagunja-Tang (SGT-4), a traditional Korean herbal decoction, comprises four oriental herbs.

(*Ginseng Radix, Glycyrrhizae Radix et Rhizoma, Atractylodis Rhizoma Alba, Poria Sclerotium*) and has been medicinally used in Asian countries to enhance vital energy and tonify spleen and stomach function. SGT-4 is applied to patients with reduced physical strength, weakened immune system, and gastrointestinal diseases [9]. Recent studies have shown that SGT-4 exhibits several effects, including immune stimulatory activity, a radioprotective effect and a soothing effect [10–12]. Like the mechanism by which vitamin C effectively promotes iPSC production, SGT-4, an antioxidant, can be a potential candidate to promote cell reprogramming by reducing the production of reactive oxygen species (ROS) during reprogramming [13]. In this study, we examined whether SGT-4 exert any effect on iPSC generation, identifying that ROS level is decreased during reprogramming process.

## Methods

### Plant materials

The four medicinal components of SGT-4 were purchased from Omniherb (Yeongcheon, Korea) and HMAX (Jecheon, Korea) in February 2008. The origin of each component was confirmed by Prof. Je Hyun Lee, (College of Oriental Medicine, Dongguk University Gyeongju, Korea) and Prof. Young-Bae Seo, (College of Oriental Medicine, Daejeon University, Daejeon, Korea). A voucher specimen (2008-KE24–1 ~ KE24–4) has been deposited at the K-herb Research Center (Korea Institute of Oriental Medicine).

### Preparation of SGT-4

The four medicinal herbs (*Ginseng Radix, Glycyrrhizae Radix et Rhizoma, Atractylodis Rhizoma Alba,* and *Poria Sclerotium*) were combined in equal amounts to produce SGT-4. The mixture was then extracted in a 10-fold volume of distilled water at 100 °C for 2 h using an electric extractor (COSMOS-660; Kyungseo Machine Co., Incheon, Korea). The solution was filtered using a standard sieve (No. 270, 53 μm; Chung Gye Sang Gong Sa, Seoul, Korea), evaporated to dryness at 40 °C under a vacuum (Eyela N-11, Tokyo, Japan), and then freeze-dried (PVT100, IlShin Bio Base, Yangju, Korea). The amount of water extract was 446.0 g (yield: 22.3%).

### High-performance liquid chromatography (HPLC) analysis of SGT-4

HPLC analysis of the extract was performed using a Shimadzu Prominence LC-20A series (Kyoto, Japan) coupled with a photodiode array (PDA) detector. The data were acquired and processed using LabSolution software (version 5.54 SP3). Seven analytes were separated on a Waters SunFire C18 column (250 × 4.6 mm, 5 μm, Milford, MA, USA) at an oven temperature of 40 °C. The mobile phases consisted of 0.1% (*v*/v) TFA in distilled water (A) and acetonitrile (B). The gradient flow was as follows: 10–60% B for 0–30 min, 60–100% B for 30–40 min, 100% B for 40–45 min, 100–10% B for 45–50 min, and 10% B for 50–60 min, with a flow-rate of 1.0 mL/min and an injection volume of 10 μL. For the simultaneous HPLC analysis of the seven primary biomarker compounds in SGT-4 sample, 200 mg of lyophilized SGT-4 extract was dissolved in 20 mL of 70% methanol and then extracted using an ultrasonicator for 20 min. The extracted solution was filtered through a 0.2 μm syringe filter (PALL Life Sciences, Ann Arbor, MI, USA) prior to HPLC analysis.

### Cell culture

Human foreskin fibroblast (HFFs) were obtained from System Biosciences (Mountain View, CA, USA) and maintained in DMEM supplemented with 10% FBS. The human dermal fibroblast (HDF) cell line was obtained from ScienCell (Carlsbad, CA, USA). HDFs were propagated in fibroblast medium supplemented with 5% FBS, 1% fibroblast growth supplement, and 1% penicillin and streptomycin (P/S). In addition, the established hiPSCs, were maintained in mTeSR 1 basal medium containing supplements on Matrigel-coating plates purchased form STEMCELL Technologies (Vancouver, BA, Canada). To maintain the hiPSCs in an undifferentiated state, the medium was refreshed daily, and the cells were passaged once a week. The cells were maintained at 37 °C in a humidified environment containing 5% CO_2._

### UVB radiation

Cells were preincubated with the indicated concentration pre-incubated with SGT-4 for 24 h, washed with PBS and exposed to a UVB (100 mJ/cm^2^) light source for 20 min (VL-6.LM, Viber Loumat, France). Afterwards, the cells were washed with PBS, and DMEM was added for 60 min.

### Derivation of hiPSCs

pMX-based retroviral vectors containing the genes for human Oct4, Sox2, Klf4, and cMyc (Addgene Inc., Cambridge, MA, USA) were transfected into HFF cells using the Neon® Transfection System (Invitrogen, Carlsbad, CA, USA). Beginning either 48 or 72 h after transfection, the medium was changed daily until hESC-like colonies emerged. To establish hiPSC cells, the colonies were treated with ReLeSR to detach the cells and transferred onto Matrigel-coated plates. Each colony was further maintained and expanded for subsequent experiments.

### Analysis of hiPSCs by flow cytometry

pMX-based retroviral vectors containing the genes for human Oct4, Sox2, Klf4, and cMyc were transfected into HFF cells. And then 48 h after transfection, the medium was changed daily until hESC-like colonies emerged. To establish hiPSC cells, we harvested differentiated from hiPSCs using 0.25% trypsin/EDTA for 2–3 min at 37 °C and resuspended in PBS/1% FBS at 1 × 10^6^ cells per 500 μl. The cells were labeled with antibodies against SSEA1-FITC, SSEA3-FITC, and SSEA4-FITC (all from Santa Cruz Biotechnology), at 4 °C for 30 min, washed twice with PBS/1% FBS, and analyzed using a FACSCanto™II flow cytometer (BD Biosciences, Bedford, MA, USA) according to the manufacturer’s instructions. The data were analyzed using BD FACSDiva (BD Biosciences).

### ATP measurement

hiPSCs were treated with different concentrations (0.25,0.5, 1, and 2 μg/ml) in a 96-well white plate for 24 h, and then total intracellular ATP content was determined using an ATP luminescent detection kit (PerkinElmer, Waltham, MA, USA) and a luminometer (Berthold Technologies, Bad Wildbad, Germany). The ATP content was determined by running an internal standard and is expressed as a percentage of the ATP content in untreated cells.

### Determination of GSH

HFFs were treated with different concentrations (0.25,0.5, 1, and 2 μg/ml) in a 6-well plate for 24 h, and then total intracellular glutathione (GSH) content was determined using a chemical assay kit (Cayman Chemical, Ann Arbor, MI, USA) following the manufacturer’s instructions.

### Measurement of ROS generation

After the UVB-irradiated cells rested for 30 min in DMEM, they were washed with PBS and incubated with a DCF-DA (10 μM) staining solution for 10 min at 37 °C in the dark. as previously described [14].

### RT-PCR

To quantify gene expression in hiPSCS, RT-PCR analysis was performed (ABI QuantStudio™ 6 Flex Real-Time PCR system, Applied Biosystems, Waltham, MA, USA) using a TaqMan universal PCR Master Mix (Applied Biosystems, USA). The relative mRNA expression levels of Nanog, Sox2, Oct4, c-Myc and KIf4 were normalized to those of GAPDH [15]. The PCR protocol comprised of 40 cycles of 95 °C for 15 s and 60 °C for 1 min. Each sample was assayed in triplicate and the relative mRNA expression levels were calculated using the ΔΔCt method.

### Western blot

Cell lysates from HFFs (5 × 10^6^) in 1× Laemmli lysis buffer (2.4 M glycerol, 0.14 M Tris (pH 6.8), 0.21 M sodium dodecyl sulfate, and 0.3 mM bromophenol blue), and boiled for 10 min. The protein content was measured using the BCA protein assay reagent (Pierce, Waltham, MA, USA). Protein samples (20 μg) were diluted in 1× lysis buffer, separated by electrophoresis (4.5%–15% gradient), and transferred onto polyvinylidene fluoride membranes (PVDF). The membranes were then incubated with primary antibodies against SOD1 (1:1000), catalase (1:1000), GPX1 (1:1000), and β-actin (1:1000) overnight followed by incubation with horseradish peroxide-conjugated secondary antibodies, Bound antibodies were detected using an enhanced chemiluminescence detection system from Amersham Bioscience (Buckinghamshire, UK). The protein expression levels were determined by analyzing the captured signals on an image analyzer (Las-3000, Fujifilm, Tokyo, Japan).

### Immunofluorescence staining

The iPSCs generated from HFFs were washed with PBS and fixed in 4% formaldehyde at room temperature (RT) for 15 min. The cells were permeablized in 0.1% Triton X-100 in PBS and blocked with a blocking solution (4% bovine serum albumin (BSA) for 1 h at RT. Primary antibodies against Oct4, Nanog, Sox2, and β-actin (Abcam, all 1:500) were diluted in blocking solution and incubated with the prepared cells overnight at 4 °C. After washing the cells with phosphate-buffered saline containing 0.01% Tween-20 (PBST), the cells were incubated with the Alexa Fluor 488- or Alexa Fluor 594-congugated secondary antibodies for 45 min at RT in the dark. The cells were washed three times with PBST, and incubated with DAPI (300 ng/ml) for 10 min to counterstain the cells nuclei. The samples were analyzed using a FluroView confocal microscopes (Olympus, Tokyo, Japan).

### Statistical analysis

The data are presented as the mean ± standard deviation (SD) of at least three separate experiments. Comparisons between two groups were made using the Student’s *t*-test in Graph Pad software (San Diego, CA, USA); and statistical significance was established at *P* < 0.05.

## Results

### Identification and quantification of the primary components of SGT-4

**Fig. 1:**
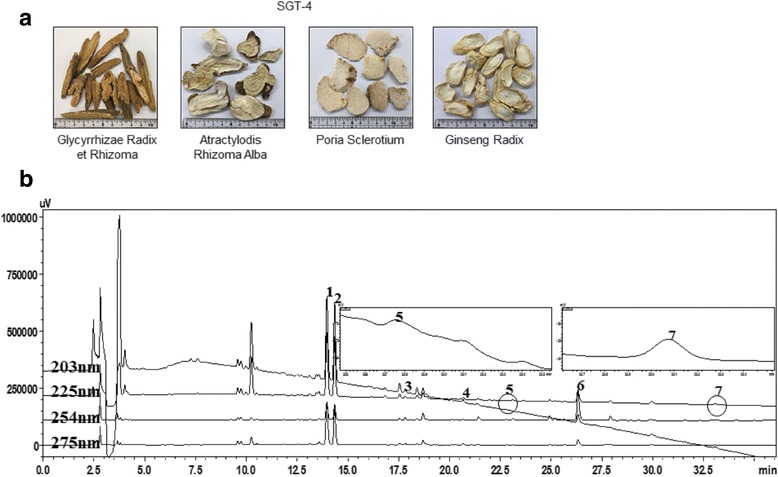
Composition of Sagunja-tang (SGT-4) and HPLC analysis of the SGT-4 decoction. **a** The SGT-4 extract was mixed from the four medicinal herbs *(Ginseng Radix, Glycyrrhizae Radix et Rhizoma, Atractylodis Rhizoma Alba,* and *Poria Sclerotium*) **b** The retention times of the 7 marker compounds-liguiritin apioside, liquiritin, ginsenoside Rg1, ginsenoside Rb1, glycyrrhizin, liquiritigenin, and atracetylenolide III

SGT-4 comprises of four herbs (*Ginseng Radix, Glycyrrhizae Radix et Rhizoma, Atractylodis Rhizoma Alba,* and *Poria Sclerotium*: Fig. 1a). Based on this composition, we selected 7 components -liquiritin apioside, liquiritin, ginsenoside Rg1, liquiritigenin, ginsenoside Rb1, glycyrrhizin, and atractylenolide III -as the primary molecules in SGT-4. The retention times of these components were 13.9, 14.33, 17.83, 20.66, 22.78, 26.31, and 33.07 min, respectively (Fig. 1b). Regarding the quantitative analysis, the analytes were detected at 203 nm for (ginsenosides Rb1 and Rg1), 225 nm (atractylenolide III), 254 nm for (glycyrrhizin), and 275 nm (liquiritin apioside, liquiritin, and liquiritigenin). The concentrations of the seven biomarker components in SGT-4 were as follows (mg/g): liquiritin apioside, 9.55; liquiritin, 3.54; ginsenoside Rg1, 5.08; liquiritigenin, 0.17;ginsenoside Rb1, 0.86; glycyrrhizin, 16.56; and atractylenolide III, 0.17.

### Optimization and characterization of hiPSCs from HFFs

**Fig. 2:**
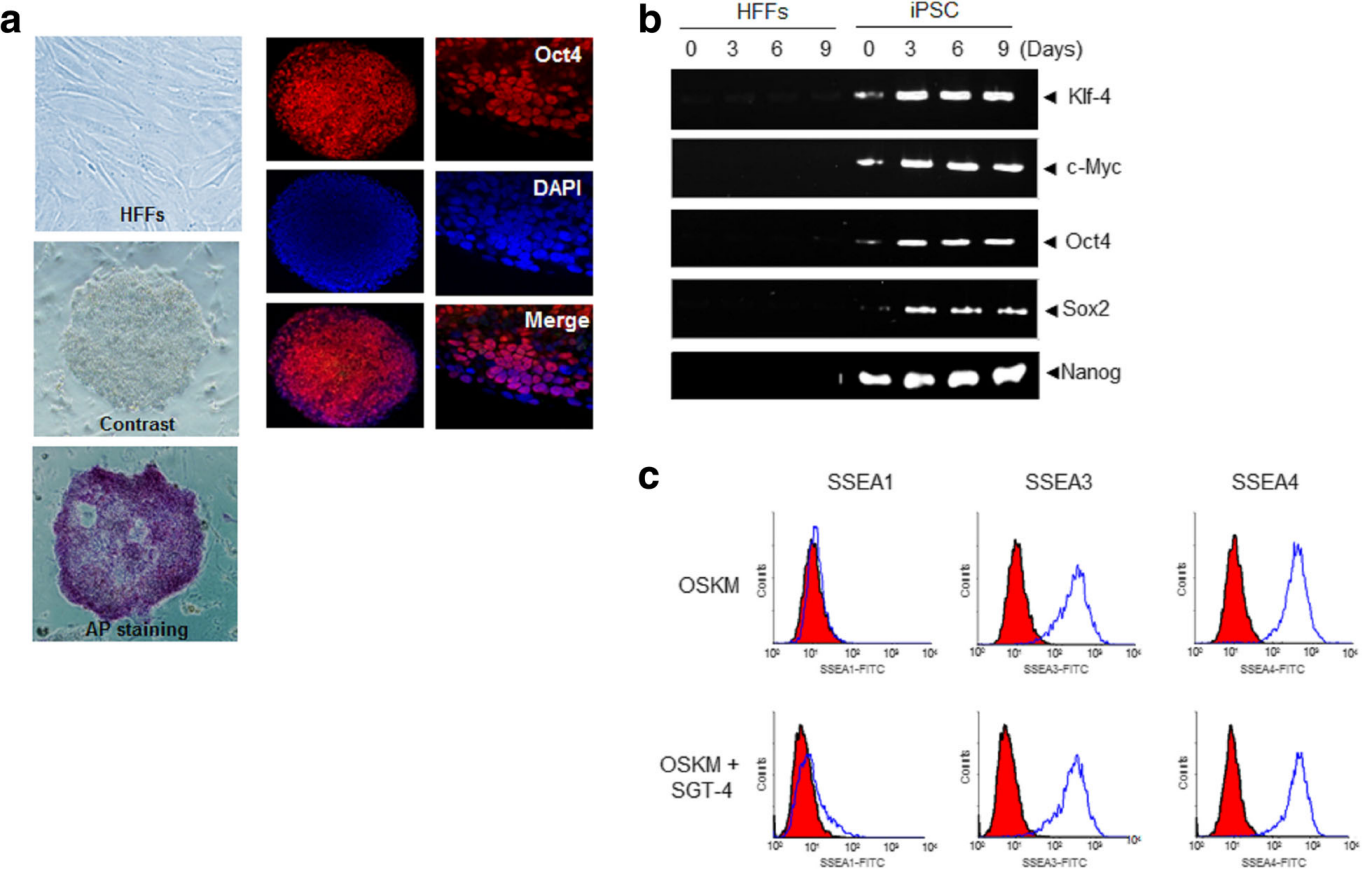
Generation and characterization of HFF-derived hiPSCs. **a** Typical morphology of human foreskin fibroblast (HFFs). Phase contrast, AP staining and immunostaining of the pluripotent-associated marker Oct4; Counter-staining with DAPI. **b** RT-PCR results demonstrated that three randomly selected 2F HFF-iPSC colonies expressed the pluripotent marker genes KIf4, c-Myc, Oct4, Nanog, and Sox2. **c** FASC analysis of the colonies differentiated into HFF-derived iPSCs in the present of SGT-4 on day 9 after transfection hiPSC markers including SSEA1, SSEA3, and SSEA4 antibodies

We examined the reprogramming of HFFs into hiPSCs using various combinations of the four core reprogramming factors (OSKM). Alkaline phosphatase positive (AP^+^) colonies derived from HFFs emerged within two weeks after transfection. Under the optimal reprogramming conditions, the AP^+^ hiPSCs-like colonies were stained to quantify the levels of the OSKM reprogramming factors, and were quantified by pluripotency marker genes, such as Klf-4, c-Myc, Oct4, Sox2, and Nanog (Fig. 2 a and b). And, we identified the hiPSCs enriched by pluripotency cell surface markers using flow cytometric analysis (Fig. 2c). Based on the results, we successfully generated HFF-derived iPSCs using four OSKM factors.

### SGT-4 promotes iPSC generation

**Fig. 3:**
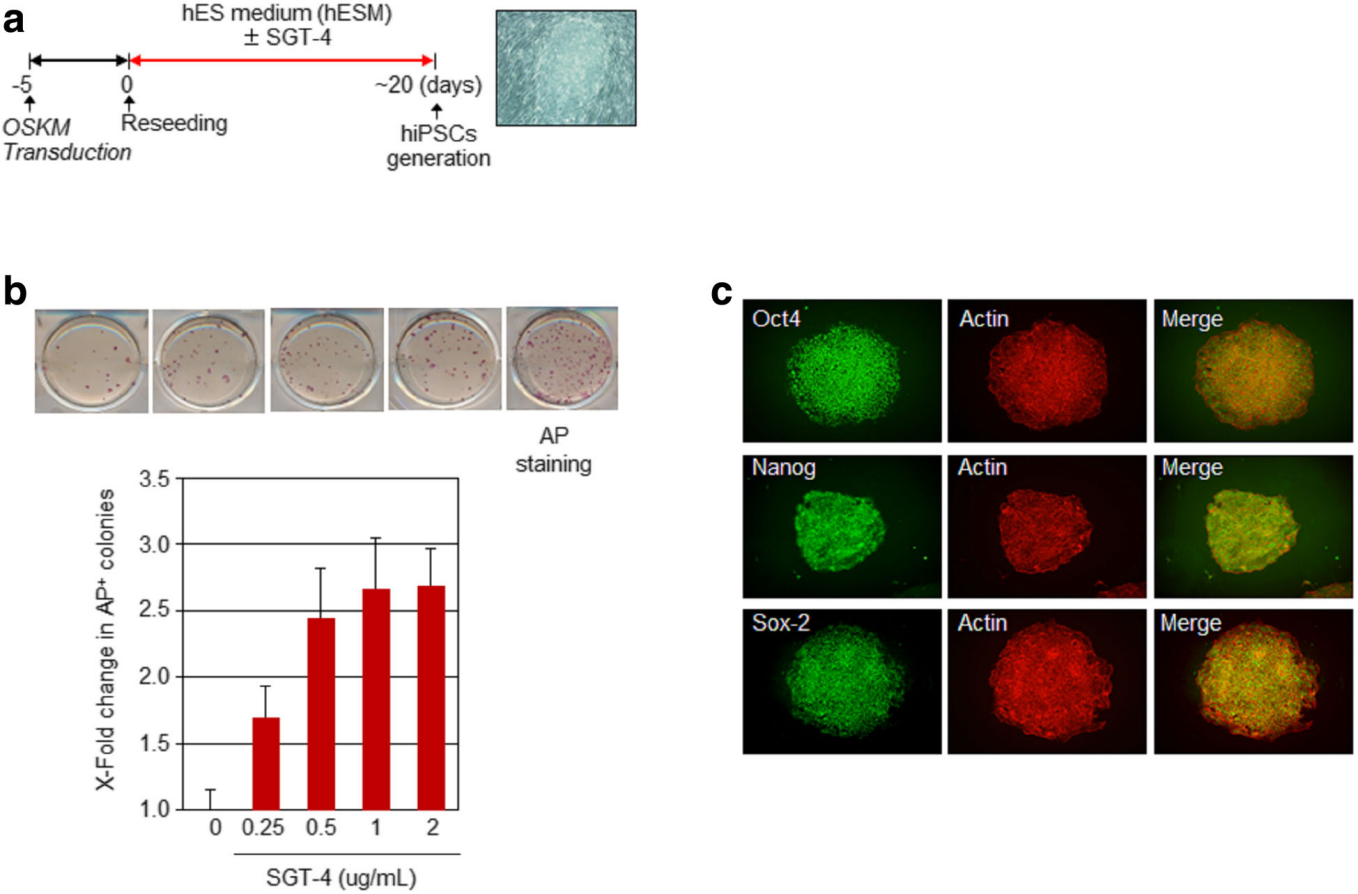
SGT-4 promotes hiPSC generation. **a** and **b** Estimated timeline of hiPSC generation and the relative number of generated AP^+^ colonies. After the cells were transfected with OSKM, they were maintained in HFF medium for 5 days, after, which they were treated with one of multiple doses of SGT-4 and transferred into human embryonic stem cells medium. The images highlight the comparison of the number of AP^+^ colonies generated from HFFs. **c** Immunostaining of the pluripotent-associated markers (Oct4, Nanog, and Sox-2) and counterstaining with β-actin antibodies

To determine whether SGT-4 affects iPSC generation, HFFs were reprogrammed using various combinations of the four core reprogramming factors (OSKM), in the presence of multiple concentrations of SGT-4. As depicted in Fig, 3a, the effect of SGT-4 on reprogramming was monitored for a period of 20 days after transfection. In the presence of SGT-4, AP^+^ colonies with ES cell-like morphology were observed at day 7 after transfection (Fig. 3). At 12 days, we observed a dose-dependent enhancement of the reprogramming efficiency when SGT-4 was administered at concentration between 0.25 and 2 μg/ml. Also, we confirmed an enrichment of pluripotency cell surface markers through flow cytometric analysis (Fig. 2c).

### SGT-4 accelerates iPSC generation

**Fig. 4:**
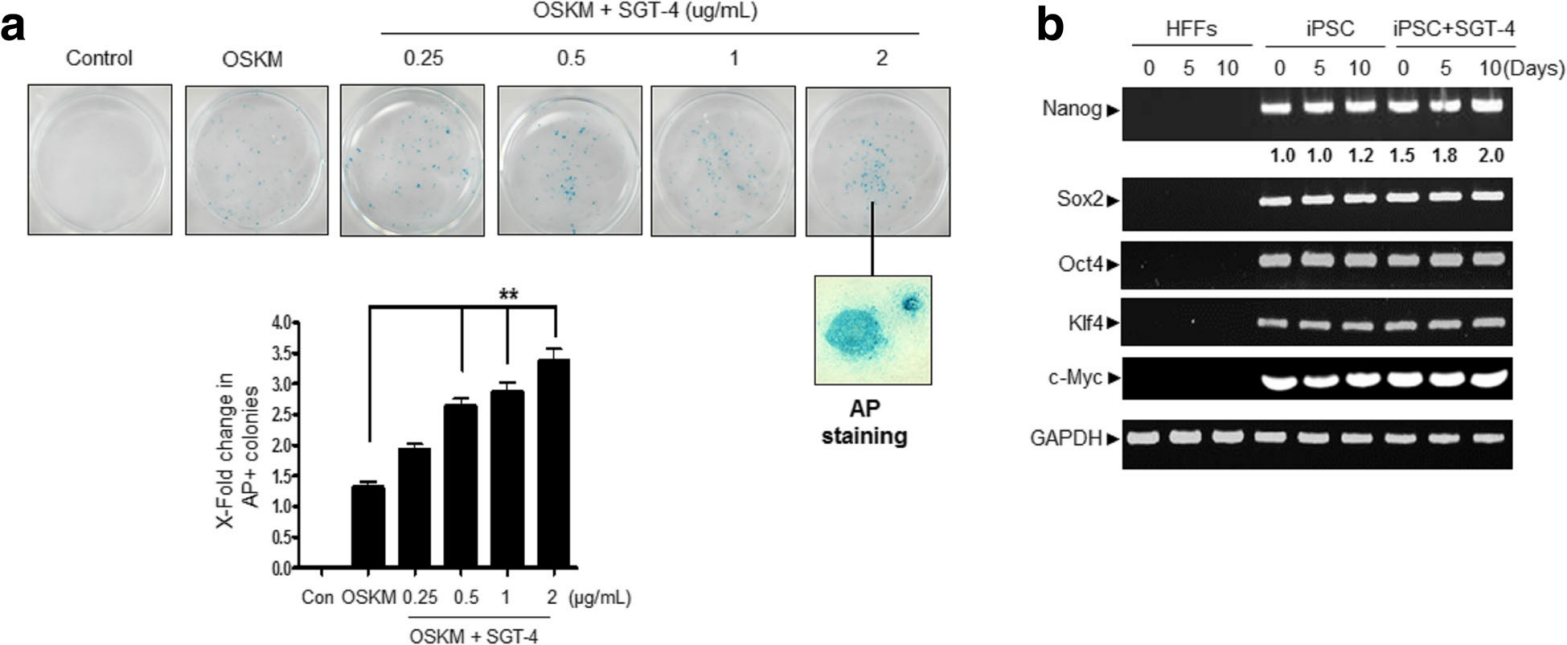
hiPSCs have an ES cell-like property. **a** Representative pictures of hiPSCs line derived in the presence of SGT-4. hiPSCs lines derived from 4F reprogramming in the presence of SGT-4 were cultured in ES cells medium on Matrigel-coated plates in the absence of feeder cells. The diagram shows the comparison of the number of AP+ colonies generated from HFFs. Each value is expressed as the mean ± SD (*n* = 3). **P < 0.01 indicates a significant difference versus OSKM treatment alone. **b** RT-PCR demonstrating that three randomly selected 4F HFF-derived iPSCs express the pluripotent marker genes Nanog, c-Myc, Sox-2, Oct4, and KIf4

To further characterize the effect of SGT-4 on iPSC generation, HFFs were reprogrammed using various combinations of the fore core reprogramming factors (OSKM) in the presence of multiple concentrations of SGT-4 (Fig. 4a). To characterize iPSCs generated by SGT-4 treatment, we identified the expression level of endogenous stem cell marker genes, Nanog, Sox2, Oct4, and Klf4 as well as c-Myc as pluripotency-related genes (Fig. 4b). Collectively, these results indicate that HFF-derived iPSCs produced in the presence of SGT-4 have pluripotency potential.

### SGT-4 blocks UVB-induced ROS generation by antioxidant enzymes expression

**Fig. 5:**
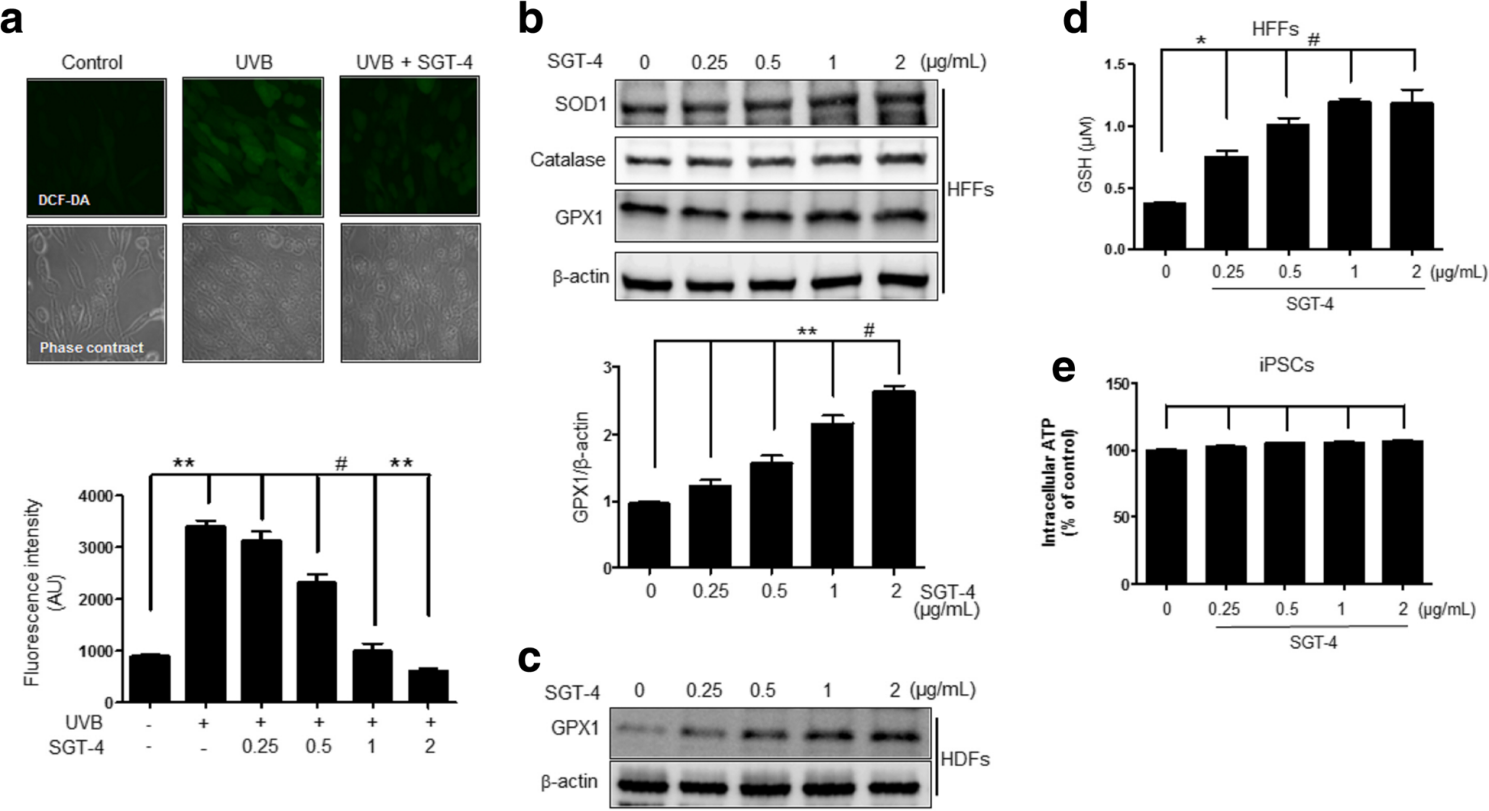
SGT-4 blocks UVB-induced ROS generation by activating antioxidant enzymes. **a** Cells were incubated with indicated concentration of SGT-4 for 24 h and then washed with PBS. The cells were then exposed to a UVB (100 mJ/cm^2^) light source for 20 min, and stained with DCF-DA for 10 min. Intracellular ROS production was analyzed using confocal microscopy. **b** HFFs were treated with various doses of SGT-4 for 18 h to determine the expression levels of SOD1, catalase, and GPX1 using Western blot analysis. **c** Human dermal fibroblasts (HDFs) were treated with various doses of SGT-4 for 18 h to determine the expression levels of GPX1 using Western blot analysis (**d**) HFFs were treated with indicates doses of SGT-4 for 24 h to determine the expression levels of glutathione (GSH) assay kit. **e** hiPSCs were incubated with indicated concentration of SGT-4 for 24 h and then ATP levels were measure by using ATP luminescent detection kit

We examined the effects of SGT-4 on cellular antioxidant activity by measuring the effects on intracellular ROS generation using the DCF-DA fluorescence staining solution. UVB radiation that can produce a large amount of ROS, caused a significant increase in ROS generation in HFF cells, whereas SGT-4 treatment inhibited UVB radiation-induced intracellular ROS generation effectively (Fig. 5a). The enhancing actions of SGT-4 on these antioxidant enzymes may contribute to its beneficial effects against cell damage, suggesting that SGT-4 has antioxidant activity. We further examined whether the ROS scavenging activity of SGT-4 was mediated by the antioxidant enzymes, such as catalase, SOD1, GPX1, and GSH. SGT-4 treatment had a positive effect on the activity of antioxidant enzymes in a concentration-dependent manner. (Fig. 5b, c, and d). Meanwhile, ATP level was measured to determine if ROS reduction by SGT-4 was the result of SGT-4 affecting the reduction of cell energy production. The ATP level was not significantly fluctuated depending on the concentration of SGT-4, indicating that SGT-4 remarkably has no effect on the energy production of iPSCs (Fig. 5e).

## Discussion

SGT-4, a traditional herbal formula comprising four different herbal medicines, (*Ginseng Radix, Glycyrrhizae Radix et Rhizoma, Atractylodis Rhizoma Alba,* and *Poria Sclerotium*) has been used in Korea and China to treat diabetes, and metabolic syndrome. In addition, it has antioxidant properties [16]. These four herbal medicines exhibit a reproductive effect in mice [17], a stimulatory effects on splenocytes [18], anti-melanogenesis activity against melanoma cells [19], and the ability to extend the lifespan of *C. elegans* [20]. In the present study, we analyzed 7 marker compounds found in an SGT-4 ginsenoside Rg1, ginsenoside Rb1, atractylenolide III, liquiritigenin, liquiritin apioside, liquiritin, and glycyrrhizin using HPLC analysis (Fig. 1b). Ginsenosides Rb1 and Rg1 promote the proliferation of primary neuron cells [21]. Atractylenolide III, and liquiritigenin have been shown to exert anti-inflammatory effects [22, 23], and glycyrrhizin attenuates stem cells- like phenotypes in papilla cells [24]. Additionally, SGT-4 is used to treat patients with reduced physical strength, a weakened immune system or gastrointestinal diseases [9].

Reprogramming of adult somatic cells into pluripotent stem cells is considered as an attractive source of stem cells for basic biological studies and regenerative medicine. The generation of hiPSCs from somatic cells with specific transcription factors allows for the possibility to produce patient-specific ES-like stem cells for therapeutic purposes [25, 26]. Consistent with these studies, our data showed that iPSCs can be generated from adult HFFs or other somatic cells by retroviral transduction of the same four transcription factors commonly expressed in ES cells: KIf-4, c-Myc, Oct4, Sox2, and we also established HFFs-derived iPSCs. The human iPSCs are similar to human embryonic stem cells in many aspects, including morphology, proliferation, self-renewal, differentiation and gene expression profile.

SGT-4 treatment facilitates iPSCs generation, enhancing the generation of AP^+^ iPSCs by 3-fold when all four factors were used for reprogramming (Fig. 4a). A recent study by Chiou et al. demonstrated that hiPSCs present low levels of cellular ROS and elevated expression of antioxidant genes such as MnSOD, Cu/ZnSOD, catalase, and GPX [27]. Our results showed that SGT-4 markedly reduced ROS generation and increased protein levels of SOD1, catalase, GPX1, and GSH activity (Fig. 5), which is consistent with the earlier finding by Chiou. Low ROS levels are necessary for the maintenance of self-renewal in several types of stem cells, including hematopoietic stem cells [28]. Similarly to the many kinds of herbal medicine have the antioxidant property, the most reason that SGT-4 markedly decrease the ROS production, would result from the seven primary compounds of SGT-4, such as ginsenoside Rg1, ginsenoside Rb1, atractylenolide III, liquiritigenin, liquiritin apioside, liquiritin, and glycyrrhizin. These major agents commonly have the antioxidant property. Thus, as some antioxidant material, for instance Vitamin C, promotes somatic cell programming in general [29–34]. For this reason, we could predict that SGT-4 additionally supports the hiPSCs generation via ROS reduction, which is essentially required for cellular reprogramming. However, we did not measure ROS levels or the changes in the expression of antioxidant proteins required to modulate stem cells fates still need to be defined during the time of SGT-4 treatment, and did not reported the mechanism by which SGT-4 exerts its activity. According to previous report, vitamin C remarkably accelerates the reprogramming efficiency as an antioxidant, not less than 100-fold, inhibiting p53 activity as reprogramming key mechanism [13]. In this context, we assume that SGT-4 may also contribute to hiPSCs generation through p53 pathway inhibition. More research to explore the effect of SGT-4 on the early reprogramming process at the molecular level as well as to better understand the technical aspects of this mechanism is required. Together, our findings indicate that the SGT-4 herbal formula facilitates the reprogramming of HFFs by antioxidant mechanism into hiPSCs in the presence of four exogenous reprogramming factors.

## Conclusion

In conclusion, our results showed that SGT-4, herbal formula facilitates the reprogramming of HFFs by antioxidant property in the presence of four exogenous reprogramming factors.
